# Timed “Up & Go” Dual-Task Tests: Age- and Sex-Specific Reference Values and Test–Retest Reliability in Cognitively Healthy Controls

**DOI:** 10.1093/ptj/pzab179

**Published:** 2021-07-17

**Authors:** Hanna B Åhman, Lars Berglund, Ylva Cedervall, Vilmantas Giedraitis, Kevin J McKee, Erik Rosendahl, Anna Cristina Åberg

**Affiliations:** Department of Public Health and Caring Sciences, Geriatrics, Uppsala University, Uppsala, Sweden; Department of Public Health and Caring Sciences, Geriatrics, Uppsala University, Uppsala, Sweden; School of Health and Welfare, Dalarna University, Falun, Sweden; Department of Public Health and Caring Sciences, Geriatrics, Uppsala University, Uppsala, Sweden; Department of Public Health and Caring Sciences, Geriatrics, Uppsala University, Uppsala, Sweden; School of Health and Welfare, Dalarna University, Falun, Sweden; Department of Community Medicine and Rehabilitation, Physiotherapy, Umeå University, Umeå, Sweden; Department of Public Health and Caring Sciences, Geriatrics, Uppsala University, Uppsala, Sweden; School of Health and Welfare, Dalarna University, Falun, Sweden

**Keywords:** Cognitive Function, Dual-Task, Reference Values, Reliability, Timed Up & Go

## Abstract

**Objective:**

The purpose of the study was to establish reference values for the Uppsala-Dalarna Dementia and Gait (UDDGait) Timed “Up & Go” dual-task (TUGdt) test variables in cognitively healthy adults and to assess these variables’ test–retest reliability.

**Methods:**

For reference values, 166 participants were recruited with approximately equal numbers and proportions of women and men in the age groups 50 to 59, 60 to 69, 70 to 79, and 80+ years (mean age = 70 years, age range = 50–91 years, 51% women). For reliability testing, 43 individuals (mean age = 69 years, age range = 50–89 years, 51% women) were recruited. Two dt tests were carried out: TUGdt naming animals and TUGdt months backward, representing 8 test variables: time scores, costs (the relative difference between single-task and dt time scores), “number of animals,” “number of months,” “animals/10 seconds ,” and “months/10 seconds .” Reference ranges for the variables were established by quantile regression in age- and sex-specific groups. For reliability, intraclass correlation coefficients (ICCs), standard error of measurement, minimal detectable change, and Bland–Altman plots were used.

**Results:**

Reference values for the TUGdt test variables are presented for the 2.5th and 97.5th percentiles. The reliability of TUGdt time scores was excellent (ICCs between 0.85 and 0.86). “Number of animals” and “animals/10 seconds” as well as “months/10 seconds” showed fair to good levels of reliability (ICCs between 0.45 and 0.58), whereas the reliability for both cost measures and “number of months” was poor (ICCs between 0.34 and 0.39).

**Conclusion:**

Normative reference values, potentially useful for clinical and research purposes, were presented in 4 age- and sex-specific groups from 50 years and older. Reliability for the test variables varied between poor and excellent, the lower estimates partly explained by some variables being the ratio of 2 other variables. In UDDGait, TUGdt tests are intended for diagnostic and predictive purposes, for which these tests are promising and require further investigations.

**Impact:**

Normative reference values and test–retest reliability results for the UDDGait TUGdt test variables were presented. These results should be useful for both clinical and research purposes.

## Introduction

Dementia is an important and growing global public health concern.^1^ Currently available methods for identifying dementia are costly and time-consuming, and new tools have been called for.^2^ In an increasing number of studies, dual-task (dt) tests that involve simultaneous performance of a mobility task and an attention-demanding verbal task have been investigated as potential tools for diagnosing or predicting dementia.^3–5^ A reduced dt ability in old age may be the result of normal age-related changes^6^^,^^7^ and/or early pathological alterations in cognition,^3^^,^^8^ in both cases possibly related to declining attentional resources or an executive dysfunction.^9^^,^^10^

The main aim of our ongoing Uppsala-Dalarna Dementia and Gait study (UDDGait)^11^ is to explore the possibility of using dt testing in the early detection of dementia. We use dt tests based on the mobility test Timed “Up & Go”^12^ (TUG) and a verbal task: (1) TUGdt NA, and (2) reciting months in reverse order (TUGdt MB). Our results have brought about a particular focus on the test variable “animals/10 s” (ie, the number of different animals named per 10 s of TUGdt NA). “Animals/10 s” correlates with concentrations of the Alzheimer disease cerebrospinal fluid biomarkers *t*-tau and *p*-tau among participants undergoing memory assessment^13^ and has a high discriminative capacity for differentiating between groups of individuals with dementia, mild cognitive impairment, subjective cognitive impairment, and healthy controls.^14^ Most importantly, animals/10 s has shown an excellent capacity for predicting dementia incidence in younger participants (<72 years) with subjective or mild cognitive impairment.^15^ To enable further interpretation of our results as well as to explore the clinical usefulness of TUGdt tests, normative reference values and investigations of reliability are required.

Normative reference values are useful clinical tools because they allow for an objective detection of deviating test performances. To be used as tools in the interpretation of test results, reference values should be calculated from a sample of individuals whose characteristics match those of the individual in question, and the testing procedures should be alike.^16^

Test–retest reliability—that is, the consistency of test results from one time to another—is central both in clinical work and in research. In repeated measurements, variability is to be expected due to biological error (differences in daily features of the test person) and technical error (differences in measurement procedures).^17^ Such errors may be systematic (eg, learning or fatigue effects, failing equipment) or random (eg, biological or mechanical variations). The reliability estimated in a test–retest setting indicates if a change in an individual’s test performance represents a real change in status or if it is caused by random variability.

To our knowledge, only 1 dt study has presented normative reference values, in which TUG was performed as quickly and safely as possible while counting aloud backward in threes from 100.^18^ However, those reference values are useful for only that specific test and not for other dt tests with other prerequisites. Regarding reliability, several dt tests have been investigated, where test–retest reliability for dt gait speed or time scores has been found to be fair to excellent among cognitively healthy controls.^18–23^ Measures of dt cost (ie, the relative difference in time or gait speed between single-task and dt performance) and verbal performances, however, have shown lower levels of reliability.^19^^,^^22^ The original single-task tests used as components in the UDDGait TUGdt NA and MB have previously been investigated for reliability. The test–retest reliability of the original single-task TUG is high among younger and older cognitively healthy adults.^20^^,^^23^^,^^24^ The test–retest reliability of the verbal fluency test naming animals is moderate to good for number of animals produced during 60 s,^25^^,^^26^ and the months backward test has excellent reliability for duration and number of errors.^27^^,^^28^

Our aims in this current study were to establish clinically useful age- and sex-specific reference values for the UDDGait TUGdt NA and TUGdt MB variables among cognitively healthy adults and to assess the test–retest reliability for these variables.

## Methods

We conducted an observational study with (1) a cross-sectional design to investigate normative reference values for TUGdt NA and MB variables, and (2) a repeated-measures design to assess these variables’ test–retest reliability. When applicable, we referred to the Consensus-Based Standards for the Selection of Health Measurement Instruments (COSMIN) guidelines.^29^^,30^ The Regional Ethical Review Board in Uppsala approved this study, and informed consent was obtained from all participants prior to study commencement.

### Participants

Participants were recruited through flyers and advertisements in local papers in Uppsala, Sweden, and Swedish-speaking Åland, Finland. For reference values, 166 individuals were recruited in Uppsala. Inclusion criteria were as follows: 50 years or older, no awareness of cognitive decline, ability to rise from a chair and walk 3 m back and forth without the use of walking aids, no need of an interpreter to communicate in Swedish, and a Mini-Mental State Examination^31^ (MMSE) score ≥27 at the time of assessment. One individual was assessed but not included due to having an MMSE score <27. The sampling was purposive, and recruitment continued until the age groups 50 to 59 years, 60 to 69 years, 70 to 79 years, and ≥80 years each comprised approximately 20 women and 20 men.

For test–retest reliability, 43 individuals were recruited. This sample comprised 21 individuals from the reference sample who agreed to participate in a retesting session. The reliability sample was completed in Åland with an additional 22 participants recruited via purposive sampling to achieve 5 or 6 women and 5 or 6 men in the age groups specified above. The inclusion criteria were the same as for the reference sample. In accordance with the COSMIN checklist,^29^ the minimum age of 50 years was set to achieve a representative sample of the main target population intended for the current TUGdt testing (ie, individuals who undergo memory assessment). Five participants were assessed but not included: 2 individuals due to an MMSE score below 27, and another 3 due to health-related issues that arose between the 2 test sessions.

### Assessments

Testing sessions for reference values as well as the first visit for reliability testing involved the same assessments as previously used in UDDGait^11^^,^^13–15^—that is, report of demographic characteristics, clinical cognitive tests^31–34^ (including MMSE and verbal fluency test), screening for depressive symptoms,^35^ TUG, TUGdt NA, TUGdt MB, and motor function tests^36–38^ (including hand grip strength and 10-m gait speed). All assessments were carried out in Swedish. For reference values, the participants were assessed at 1 session. For test–retest reliability, the participants were assessed at 2 sessions. The interval between test and retest was set at 10 (SD = 4) days, which was considered long enough for the participants’ memory of the tasks to have diminished but too short for changes in motor, cognitive, or dt abilities to occur.^29^ Most participants carried out the test and retest within the set interval. However, for practical reasons, the time interval was deviant for 6 participants (ie, 3, 4, 15, 16, 23, and 26 days between test and retest).

The retest session involved MMSE, TUG, TUGdt NA, TUGdt MB, and the motor function tests. The MMSE was carried out before the dt testing, both to enhance the comparability of the test and retest sessions, and again as an inclusion criterion (score of ≥27). Other test conditions (eg, administration, environment, instructions) were held constant as far as possible. Additionally, to ensure that the participants were stable between the test and the retest session, they were asked to report any incidents or changes in health that could affect their performance.^29^

The UDDGait TUGdt testing has been described previously.^11^ The dt testing was preceded by the single-task TUG. The TUG test involves measuring the time required for a test person to carry out a movement sequence at a comfortable pace: to rise from a chair, walk 3 m, turn at a marking on the floor, return to the chair, and sit down again.^12^ After performing the single-task TUG, the participants carried out 2 different TUGdt tests in the following order: TUGdt NA and TUGdt MB (starting from December). The physical therapist who led the testing gave standardized instructions to the participant before each test, including instructions to complete all tests at their own speed concerning both mobility and verbal performance and to complete the mobility sequence if they did not know what to say. The tests were timed with a stopwatch to an accuracy of 0.01 second and video-recorded from frontal and lateral views.

All TUGdt test variables used in the current analyses were calculated based on time scores and/or the number of words recited (different animals or months in correct, reverse order). The variables animals/10 s and months/10 s were calculated as 10^*^(TUGdt number of words/TUGdt time score). The cost variables were calculated as 100×(TUGdt time score − TUGst time score)/TUGst time score.

### Statistical Analyses

Descriptive data were summarized by means and SDs; frequencies; percentages, medians and interquartile ranges; and minimum and maximum values, when appropriate.

In the current study, the reference values denote the percentile values that provide a 95% range for the healthy controls. By quantile regression,^39^ the 2.5th and 97.5th percentiles were estimated to define the range of 95% of the healthy controls in age- and sex-specific groups. The quantile regression method increases the power to detect differences in the upper and lower tails by weighing portions of the sample to generate coefficient estimates.^40^ Because a high proportion (73%) of the healthy controls had a university education, a sensitivity analysis was carried out where participants with and without university education were analyzed separately for comparison.

Single-measurement absolute agreement intraclass correlation coefficients (ICCs) estimated from a 2-way mixed effects (with participant as random factor and time as fixed factor) linear model were used for the test–retest reliability analyses.^41^ The variables TUGdt NA and MB time scores as well as TUGdt NA and MB cost were non-normally distributed and were therefore log transformed. Due to the cost variables having negative values, a constant, *c* = 20, was added to the original values before log transformation. Bootstrap estimation was used for the 95% CIs with 100,000 bootstrap samples by the percentile-*t* method.^42^ The standard error of measurement (SEM) was calculated as a measure of absolute reliability: SEM = *S_x_*√1 − ICC; in this equation, *S_x_* is the SD of the set of measurements. The minimal detectable change (MDC) was calculated as: 1.96 × √2 × SEM. Bland–Altman plots with median difference and limits of agreement (2.5th and 97.5th percentiles)^43^ were used to quantify possible systematic error and to check for outliers. A 95% CI for a median difference that did not cover zero was defined as a statistically significant difference. Because not all participants performed the test and retest within 10+/-4 days, a sensitivity analysis was carried out where only participants who carried out the retest within this time interval were included.

Analyses were carried out using SPSS version 27 (IBM Corp, Armonk, NY, USA), R version 3.6.3^44^ (R Core Team [2020]. R: A language and environment for statistical computing. R Foundation for Statistical Computing, Vienna, Austria. URL https://www.R-project.org/), and SAS version 9.4 (SAS Institute Inc, Cary, NC, USA).

### Role of the Funding Source

The funding source had no role in the study’s design or conduct of this study. In regards to the reporting of the study, the funders have required Open Access publication.

## Results

### Reference Sample: Participants

An overview of participants’ characteristics and test results for the reference sample is shown in Table 1. A total of 166 participants (age range = 50–91 years, 51% women) were included. The majority of the sample was married or cohabiting (71%), and 73% had a university education. These proportions were higher in the younger age groups. A sensitivity analysis found that education level did not substantially affect the TUGdt reference values. Four participants (3 men, 1 woman), equally distributed across age groups, had depressive symptoms.

**Table 1:** Characteristics and Test Results for the Reference Sample by Age and Sex (N = 166)^a^

**Characteristic**	**Value for the Following Sample**								
	**Total Sample (N = 166)**	**50–59 y**		**60–69 y**		**70–79 y**		**≥80 y**	
		**Women (n = 20)**	**Men (n = 19)**	**Women (n = 23)**	**Men (n = 20)**	**Women (n = 22)**	**Men (n = 22)**	**Women (n = 20)**	**Men (n = 20)**
Age, y									
Mean (SD)	69.5 (10.7)	55.2 (2.8)	54.7 (3.3)	65.6 (3.0)	64.9 (2.8)	73.9 (2.5)	73.8 (2.5)	83.0 (3.3)	84.0 (3.3)
Minimum–maximum	50 to 91	50 to 59	50 to 59	61 to 69	61 to 69	70 to 79	70 to 79	80 to 91	80 to 91
Married or cohabiting, no. (%)	118 (71)	18 (90)	19 (100)	15 (65)	17 (85)	10 (46)	17 (77)	6 (30)	16 (80)
University education, no. (%)	121 (73)	17 (85)	15 (79)	17 (74)	17 (85)	18 (82)	16 (73)	8 (40)	13 (65)
Depressive symptoms*^b^**,* no. (%)	4 (2)	0 (0)	1 (5)	0 (0)	1 (5)	0 (0)	1 (5)	1 (5)	0 (0)
Cognitive/motor function test result									
Verbal fluency test,*^c^* score									
Median (IQR)	23.5 (20.0 to 29.0)	26.0 (22.3 to 33.0)	28.0 (24.0 to 35.0)	23.0 (22.0 to 31.0)	29.0 (24.0 to 33.3)	25.5 (20.5 to 30.0)	22.0 (18.8 to 26.0)	20.0 (17.5 to 22.8)	20.0 (16.3 to 22.8)
Minimum–maximum	11 to 41	18 to 40	19 to 38	15 to 36	15 to 41	11 to 38	15 to 41	13 to 37	11 to 30
Hand grip, kg									
Median (IQR)	33.1 (27.2 to 44.7)	31.8 (27.4 to 34.0)	52.2 (48.5 to 57.2)	31.3 (29.0 to 34.0)	50.1 (45.5 to 53.9)	26.8 (23.8 to 29.6)	41.5 (34.4 to 48.2)	20.4 (19.1 to 23.0)	31.5 (27.4 to 36.7)
Minimum–maximum	10.9 to 74.8	17.2 to 43.1	40.4 to 74.8	21.3 to 36.3	37.2 to 64.4	13.2 to 34.0	29.9 to 61.2	10.9 to 36.3	15.9 to 54.0
10-m gait speed,*^d^* m/s									
Median (IQR)	1.3 (1.1 to 1.4)	1.4 (1.3 to 1.5)	1.4 (1.2 to 1.5)	1.3 (1.1 to 1.5)	1.4 (1.2 to 1.5)	1.2 (1.1 to 1.3)	1.2 (1.1 to 1.3)	1.1 (0.9 to 1.1)	1.1 (1.0 to 1.3)
Minimum–maximum	0.6 to 1.7	0.9 to 1.7	1.1 to 1.7	0.9 to 1.6	0.9 to 1.5	0.9 to 1.5	1.0 to 1.5	0.7 to 1.2	0.6 to 1.5
TUGst time, s									
Median (IQR)	10.1 (9.0 to 11.4)	8.9 (7.8 to 9.4)	9.5 (8.9 to 10.3)	9.6 (8.6 to 10.3)	9.7 (8.5 to 10.0)	11.3 (9.1 to 12.2)	10.1 (9.5 to 11.3)	11.6 (10.5 to 13.2)	11.9 (10.7 to 13.9)
Minimum–maximum	6.1 to 24.1	7.3 to 10.9	6.1 to 11.3	7.2 to 14.5	7.4 to 12.2	7.8 to 13.6	7.2 to 14.8	8.4 to 15.2	7.4 to 24.1
TUGdt NA test result									
NA time, s									
Median (IQR)	11.0 (9.8 to 13.8)	9.9 (8.3 to 10.3)	9.9 (9.5 to 10.7)	10.5 (9.5 to 13.3)	9.9 (8.8 to 11.1)	12.4 (9.1 to 14.7)	12.2 (10.7 to 13.2)	15.5 (12.2 to 17.5)	14.8 (13.3 to 17.4)
Minimum–maximum	5.8 to 26.7	7.4 to 13.2	5.8 to 11.9	7.3 to 14.6	7.5 to 13.7	7.8 to 22.8	8.3 to 18.2	9.7 to 24.5	7.1 to 26.7
NA cost, %									
Median (IQR)	9.9 (2.7 to 23.0)	6.7 (4.0 to 14.4)	5.8 (−4.3 to 10.3)	7.8 (0.7 to 24.4)	5.9 (0.0 to 12.0)	6.0 (−0.5 to 20.3)	14.2 (7.8 to 22.6)	22.1 (16.4 to 31.8)	20.6 (7.9 to 43.5)
Minimum–maximum	−10.2 to 100.5	−3.5 to 26.0	−7.9 to 23.0	−8.0 to 51.5	−10.2 to 32.0	−5.5 to 74.9	2.3 to 42.8	1.9 to 100.5	−4.5 to 52.1
No. of animals									
Median (IQR)	8.0 (7.0 to 9.0)	8.0 (6.3 to 8.8)	7.0 (6.0 to 8.0)	9.0 (7.0 to 9.0)	8.0 (6.3 to 9.0)	8.0 (7.0 to 10.0)	7.0 (5.8 to 8.3)	8.0 (7.3 to 11.0)	7.5 (6.0 to 9.8)
Minimum–maximum	3 to 15	6 to 11	5 to 12	7 to 12	5 to 12	6 to 15	4 to 10	5 to 13	3 to 11
Animals/10 s									
Median (IQR)	6.7 (5.7 to 8.3)	8.2 (6.3 to 10.0)	6.7 (6.0 to 8.4)	7.4 (6.6 to 9.5)	7.5 (6.6 to 9.1)	6.8 (5.9 to 8.9)	6.3 (4.9 to 7.3)	6.1 (5.1 to 7.0)	4.6 (4.0 to 6.2)
Minimum–maximum	2.1 to 12.4	5.5 to 11.3	4.9 to 12.4	5.2 to 12.1	4.7 to 10.3	3.6 to 11.6	2.8 to 9.3	3.3 to 8.5	2.1 to 9.9
TUGdt MB test result									
MB time, s									
Median (IQR)	11.1 (9.6 to 14.0)	9.6 (8.6 to 10.6)	9.8 (9.4 to 11.2)	10.1 (9.2 to 11.9)	9.7 (8.5 to 11.1)	12.5 (9.8 to 14.9)	12.2 (11.0 to 13.9)	16.0 (13.2 to 17.3)	16.1 (13.7 to 17.2)
Minimum–maximum	6.1 to 25.8	7.6 to 12.8	6.1 to 12.7	7.3 to 15.1	7.6 to 12.9	7.9 to 18.6	9.4 to 21.7	10.0 to 23.7	8.3 to 25.8
MB cost, %									
Median (IQR)	11.5 (3.1 to 28.8)	7.9 (4.2 to 13.1)	6.5 (−1.9 to 17.4)	2.4 (−4.8 to 17.1)	2.3 (−4.8 to 10.6)	11.6 (3.7 to 24.4)	18.3 (10.2 to 33.4)	29.1 (18.8 to 52.2)	25.9 (12.0 to 41.0)
Minimum–maximum	−17.9 to 113.5	−1.5 to 23.2	−9.5 to 22.3	−17.9 to 50.7	−10.3 to 17.5	−10.9 to 43.1	−7.0 to 59.1	2.4 to 113.4	5.0 to 61.3
No. of months									
Median (IQR)	9.0 (8.0 to 11.0)	8.0 (8.0 to 9.8)	8.0 (7.0 to 9.0)	9.0 (8.0 to 10.0)	9.5 (8.0 to 11.0)	9.5 (7.8 to 12.0)	8.5 (7.0 to 11.0)	11.0 (9.0 to 12.0)	10.0 (8.3 to 12.0)
Minimum–maximum	3 to 13	4 to 11	6 to 10	3 to 12	6 to 12	5 to 13	4 to 12	4 to 12	5 to 12
Months/10 s									
Median (IQR)	7.8 (6.4 to 9.4)	9.3 (7.9 to 10.4)	8.1 (6.4 to 8.5)	8.4 (7.6 to 9.8)	10.1 (8.3 to 11.1)	7.5 (6.2 to 9.6)	7.2 (6.0 to 8.2)	6.9 (5.3 to 7.4)	6.9 (4.9 to 7.6)
Minimum–maximum	2.9 to 13.3	4.4 to 12.4	4.7 to 11.6	2.9 to 12.9	5.6 to 12.8	3.4 to 13.3	3.5 to 10.7	3.6 to 9.6	3.2 to 8.7

*^a^* IQR = interquartile range; MB = months backward; NA = naming animals; TUGdt = Timed “Up & Go” dual-task; TUGst = Timed “Up & Go” single-task.

^*b*^Depressive symptoms were defined as 2 points or more on the 4-item Geriatric Depression Scale.

^*c*^The verbal fluency test involved naming as many different animals as possible in 60 s while in a sitting position.

^*d*^Habitual gait speed was measured from a static start in a 10-m corridor. Missing cases were as follows: for age group 50–59 years, 2 men; for age group 60–69 years, 1 woman; for age group 70–79 years, 1 man; and for age group ≥80 years, 1 man.

**Table 2:** Reference Values for TUGdt Test Variables in Age and Sex Groups^a^

**Sex**	**Variable**	**Test Result**	**Percentile**	**Value (95% CI) for the Following Age Groups**			
				**50–59 y**	**60–69 y**	**70–79 y**	**≥80 y**
Women	TUGdt NA	Time score, s	2.5th	6.8 (4.2 to 9.4)	7.6 (6.0 to 9.3)	8.3 (6.6 to 10.0)	9.0 (6.5 to 11.5)
			97.5th	**13.2** (11.1 to 15.4)	**17.8** (16.4 to 19.2)	**21.38** (20.0 to 22.8)	**25.4** (23.3 to 27.5)
		NA cost, %	2.5th	−10.5 (−14.9 to −6.1)	−6.1 (−8.9 to −3.3)	−2.6 (−5.5 to 0.2)	1.2 (−3.1 to 5.5)
			97.5th	**26.5** (15.7 to 37.3)	**53.3** (46.3 to 60.3)	**74.5** (67.4 to 81.6)	**98.0** (87.5 to 108.6)
		No. of animals	2.5th	**6.0** (4.8 to 7.2)	**6.0** (5.2 to 6.8)	**6.0** (5.2 to 6.8)	**6.0** (4.8 to 7.2)
			97.5th	11.1 (8.7 to 13.5)	12.7 (11.2 to 14.2)	14.0 (12.4 to 15.5)	15.4 (13.1 to 17.7)
		Animals/10 s	2.5th	**5.4** (4.6 to 6.3)	**4.6** (4.1 to 5.2)	**4.0** (3.4 to 4.5)	**3.3** (2.4 to 4.1)
			97.5th	12.5 (10.4 to 14.5)	11.7 (10.4 to 13.0)	11.1 (9.7 to 12.4)	10.4 (8.4 to 12.4)
	TUGdt MB	Time score, s	2.5th	6.6 (5.5 to 7.6)	7.7 (7.1 to 8.4)	8.7 (8.0 to 9.3)	9.7 (8.6 to 10.7)
			97.5th	**11.9** (7.9 to 15.9)	**15.6** (13.1 to 18.2)	**18.6** (16.0 to 21.2)	**22.0** (18.0 to 25.9)
		MB cost, %	2.5th	−14.0 (−24.2 to −3.5)	−11.9 (−18.7 to −5.2)	−10.3 (−17.2 to −3.5)	−8.5 (−18.7 to 1.7)
			97.5th	**20.7** (−8.3 to 49.7)	**45.1** (26.4 to 63.8)	**64.5** (45.5 to 83.4)	**85.9** (57.6 to 114.2)
		No. of months	2.5th	**4.0** (2.0 to 6.0)	**4.0** (2.7 to 5.3)	**4.0** (2.7 to 5.3)	**4.0** (2.1 to 5.9)
			97.5th	11.6 (11.4 to 11.9)	12.3 (12.1 to 12.4)	12.8 (12.7 to 13.0)	13.4 (13.2 to 13.6)
		Months/10 s	2.5th	**4.4** (2.8 to 6.0)	**4.1** (3.1 to 5.1)	**3.8** (2.8 to 4.9)	**3.5** (2.0 to 5.1)
			97.5th	13.1 (12.1 to 14.1)	12.8 (12.2 to 13.5)	12.6 (12.0 to 13.3)	12.4 (11.4 to 13.4)
Men	TUGdt NA	Time score, s	2.5th	6.3 (3.7 to 8.9)	7.2 (5.5 to 9.0)	8.1 (6.4 to 9.8)	9.0 (6.5 to 11.6)
			97.5th	**12.3** (10.1 to 14.5)	**16.3** (14.9 to 17.7)	**19.9** (18.5 to 21.3)	**23.8** (21.7 to 25.9)
		NA cost, %	2.5th	−8.2 (−12.6 to −3.7)	−6.7 (−9.7 to −3.8)	−5.5 (−8.4 to −2.6)	−4.1 (−8.4 to 0.3)
			97.5th	**26.2** (15.2 to 37.2)	**34.8** (27.5 to 42.0)	**42.4** (35.3 to 49.5)	**50.8** (40.1 to 61.5)
		No. of animals	2.5th	**5.3** (4.1 to 6.6)	**4.6** (3.7 to 5.4)	**3.9** (3.1 to 4.7)	**3.1** (1.9 to 4.3)
			97.5th	12.1 (9. to 14.5)	11.8 (10.2 to 13.4)	11.5 (9.9 to 13.0)	11.1 (8.8 to 13.5)
		Animals/10 s	2.5th	**5.3** (4.4 to 6.1)	**4.0** (3.5 to 4.6)	**2.9** (2.4 to 3.5)	**1.7** (0.9 to 2.5)
			97.5th	11.5 (9.4 to 13.6)	10.3 (9.0 to 11.7)	9.4 (8.0 to 10.7)	8.3 (6.2 to 10.3)
	TUGdt MB	Time score (s)	2.5th	6.5 (5.4 to 7.6)	7.3 (6.6 to 8.0)	8.0 (7.3 to 8.7)	8.8 (7.8 to 9.9)
			97.5th	**12.5** (8.5 to 16.6)	**16.5** (13.8 to 19.2)	**20.0** (17.3 to 23.0)	**23.8** (19.8 to 27.8)
		MB cost, %	2.5th	−15.0 (−25.6 to −4.3)	−9.4 (−16.4 to −2.4)	−4.4 (−11.3 to 2.5)	1.0 (−9.4 to 11.4)
			97.5th	**24.6** (−5.1 to 54.2)	**39.1** (19.6 to 58.6)	**52.1** (32.9 to 71.2)	**66.3** (37.5 to 95.1)
		No. of months	2.5th	**5.9** (3.9 to 7.9)	**5.6** (4.2 to 6.9)	**5.3** (4.0 to 6.6)	**5.0** (3.1 to 7.0)
			97.5th	12.0 (11.8 to 12.2)	12.0 (11.9 to 12.2)	12.0 (11.9 to 12.2)	12.0 (11.8 to 12.2)
		Months/10 s	2.5th	**4.8** (3.2 to 6.4)	**4.3** (3.2 to 5.3)	**3.8** (2.7 to 4.8)	**3.3** (1.7 to 4.8)
			97.5th	15.0 (13.9 to 16.0)	12.6 (11.9 to 13.3)	10.5 (9.8 to 11.1)	8.1 (7.1 to 9.1)

^*a*^Bold type indicates the value that is clinically useful for comparisons (ie, the 97.5th percentile for time scores and cost measures and the 2.5th percentile for number of animals, number of months, animals/10 s, and months/10 s. MB = months backward; NA = naming animals; TUGdt = Timed “Up & Go” dual-task.

### Reference Values

In Table 2, reference values are presented for the 2.5th and 97.5th percentile, where the upper and lower limits provide values with which an individual’s performance can be compared. For variables for which high values represent poorer performance (time scores and cost measures), the 97.5th percentile is clinically most relevant for comparisons and is therefore highlighted in the table. Conversely, for variables for which low values represent poorer performance (number of animals or months, and number of animals or months/10 s), the 2.5th percentile is highlighted (Tab. 2). Among both women and men, the reference ranges appeared to vary with age (Tab. 2).

### Reliability Sample: Participants

An overview of the participants’ characteristics and test results for the test–retest reliability sample are summarized in Table 3. A total of 43 participants (age range = 50–89 years, 51% women) were included, with approximately equal numbers of participants and proportions of men and women in each of the 4 age groups. A majority was married or cohabiting (77%) and had a university education (51%). None of the participants had depressive symptoms.

**Table 3:** Participant Characteristics, First-Session Cognitive/Motor Function Test Results, and Dual-Task Test Results for Test and Retest Sessions (n = 43)^a^

**Characteristic**	**Value for Reliability Sample**			
	**Mean (SD)**	**No. (%)**	**Median (IQR)**	**Minimum–Maximum**
Age, y	69.3 (11.08)			50 to 89
Women		22 (51.2)		
Married or cohabiting		33 (76.7)		
University education		22 (51.2)		
Depressive symptoms*^b^*		0 (0)		
Cognitive/motor function test result for first session				
Verbal fluency test*^c^* score			24.0 (20.0 to 29.0)	16 to 38
Hand grip, kg			34.0 (27.2 to 40.8)	17.2 to 78.4
10-m gait speed,*^d^* m/s			1.3 (1.1 to 1.4)	0.7 to 1.7
TUGst time, s			9.8 (8.9 to 11.1)	7.2 to 15.4
TUGdt NA test result for test and retest				
NA time, s				
Test			10.8 (9.9 to 12.7)	7.9 to 16.4
Retest			10.8 (9.4 to 12.9)	7.1 to 18.3
NA cost, %				
Test			10.4 (4.7 to 16.6)	−7.9 to 47.6
Retest			11.5 (3.3 to 17.0)	−5.4 to 37.4
No. of animals				
Test			7.0 (7.0 to 10.0)	2 to 12
Retest			8.0 (7.0 to 10.0)	4 to 12
Animals/10 s				
Test			7.1 (6.0 to 8.8)	1.8 to 11.3
Retest			8.0 (6.1 to 9.4)	2.2 to 12.2
TUGdt MB test result for test and retest				
MB time, s				
Test			10.8 (9.8 to 13.2)	7.6 to 20.1
Retest			10.5 (9.3 to 11.9)	6.1 to 19.0
MB cost, %				
Test			9.7 (3.6 to 18.7)	−5.7 to 59.5
Retest			9.3 (1.8 to 14.5)	−17.5 to 52.3
No. of months				
Test			9.0 (7.0 to 10.0)	5 to 12
Retest			9.0 (8.0 to 10.0)	5 to 12
Months/10 s				
Test			8.1 (6.2 to 9.1)	3.2 to 12.2
Retest			7.7 (6.6 to 10.4)	4.4 to 13.5

^*a*^IQR = interquartile range; MB = months backward; NA = naming animals; TUGdt = Timed “Up & Go” dual-task; TUGst = Timed “Up & Go” single-task.

^*b*^Depressive symptoms were defined as 2 points or more on the 4-item Geriatric Depression Scale.

^*c*^The verbal fluency test involved naming as many different animals as possible in 60 s while in a sitting position.

^*d*^Habitual gait speed was measured from a static start in a 10-m corridor.

### Test–Retest Reliability

The ICC estimate of the TUGdt NA time score was 0.86, with an SEM of 0.95 and an MDC of 2.63 (Tab. 4). The results were similar for the TUGdt MB time score, where the ICC estimate was 0.85, the SEM was 1.00, and the MDC was 2.77. The verbal performance of TUGdt NA had a higher level of reliability than TUGdt MB, as shown by number of animals (ICC = 0.57, SEM = 1.27, MDC = 3.52) compared with number of months (ICC = 0.38, SEM = 1.54, MDC = 4.27). Correspondingly, animals/10 s (ICC = 0.58, SEM = 1.33, MDC = 3.69) had a higher reliability than months/10 s (ICC = 0.45, SEM = 1.69, MDC = 4.68). Both cost measures showed low reliability (Tab. 4). The results of a sensitivity analysis including only participants who carried out the retest within 10+/-4 days were substantially similar.

**Table 4:** ICC Estimates for TUGdt Test Variables With Bootstrap 95% CIs, SEM, and MDC (n = 43)^a^

**TUGdt Test Result**	**ICC Estimate (Bootstrap 95% CI)**	**SEM**	**MDC**
NA			
Time score, s	0.86 (0.77–0.91)	0.95	2.63
NA cost, %	0.34 (0.10–0.54)	8.68	24.06
No. of animals	0.57 (0.39–0.69)	1.27	3.52
Animals/10 s	0.58 (0.37–0.74)	1.33	3.69
MB			
Time score, s	0.85 (0.74–0.92)	1.00	2.77
MB cost, %	0.39 (0.21–0.56)	11.06	30.66
No. of months	0.38 (0.10–0.60)	1.54	4.27
Months/10 s	0.45 (0.20–0.67)	1.69	4.68

^*a*^MB = months backward; MDC = minimal detectable change; NA = naming animals; SEM = standard error of measurement; TUGdt = Timed “Up & Go” dual-task.

The Bland–Altman plots presented in the Figure describe the differences between test and retest plotted against the mean value for the variables TUGdt NA time score (plot A), TUGdt MB time score (plot B), animals/10 s (plot C), and months/10 s (plot D). These plots use the variables’ original values for ease of interpretation (ie, prior to log transformation). For the variable TUGdt NA time score, the median difference was −0.03 (95% CI = −0.34 to 0.059) (Figure, plot A). Because the CI covered zero, the difference was not statistically significant. Likewise, for TUGdt MB months/10 s (Figure, plot D), the median difference (−0.31 [95% CI = −1.16 to 0.43]) was not significant. However, for TUGdt MB time score (Figure, plot B) and TUGdt NA animals/10 s (Figure, plot C), the median differences (TUGdt MB time score: 0.23 [95% CI = 0.12–0.75]; TUGdt NA animals/10 s: −0.79 [95% CI = −1.06 to −0.18]) indicated systematic error. The patterns of TUGdt NA and MB time scores revealed that a larger time score entailed greater differences between measurements (Figure, plots A and B). Outliers were identified in all plots (A: n = 2, B: n = 2, C: n = 1, D: n = 2). These participants’ video recordings were studied, and explanations of the differences between the test occasions were found. In the TUGdt NA test, the 3 participants behaved differently on 1 test occasion compared with the other, either by starting to laugh at their own verbal performance (n = 2) or by accidently pushing the chair before sitting down and then correcting its position before finalizing the test (n = 1). Regarding the TUGdt MB test, the differences between test occasions were not as apparent. Either the participant made a verbal mistake (recited an incorrect month, immediately noticed the mistake and got confused) (n = 2), was generally more hesitant when reciting the months (n = 1), or walked slower (n = 1).

**Figure:**
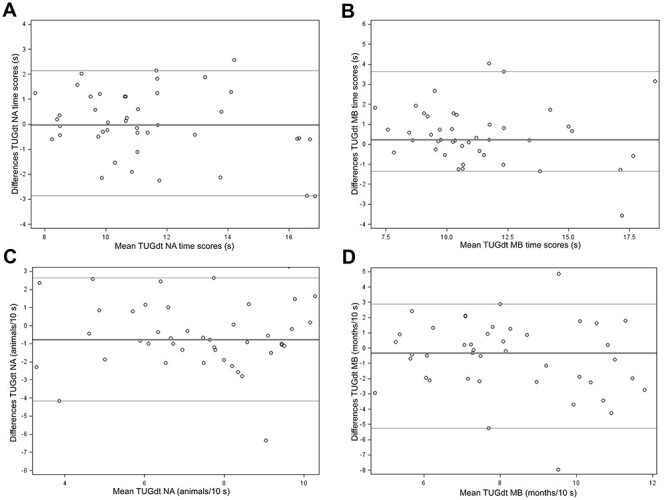
Bland–Altman plots for the Timed “Up & Go” dual-task (TUGdt) naming animals (NA) time score (A), TUGdt months backward (MB) time score (B), TUGdt NA animals/10 s (C), and TUGdt MB months/10 s (D). Reference lines represent the median difference and limits of agreement (2.5th and 97.5th percentiles of differences).

Plots concerning the variables TUGdt cost and number of animals and months are not presented here because these did not show any distinct pattern or entail systematic error: TUGdt NA cost (median difference = −1.30, 95% CI = −4.87 to 4.54), TUGdt MB cost (median difference = 1.19, 95% CI = −2.09 to 6.70), TUGdt NA number of animals (median difference = 0.00, 95% CI = −1.00 to 0.00), and TUGdt MB number of months (median difference = 0.00, 95% CI = −1.00 to 1.00).

## Discussion

The current study establishes reference values for TUGdt NA and MB test variables in age- and sex-specific groups, where the upper and lower limits indicate the range of variability in cognitively healthy controls. The test–retest ICC estimates showed that the reliability of TUGdt NA and MB time scores was excellent. The variables “number of animals” and “animals/10 s” as well as “months/10 s” showed fair to good levels of reliability, whereas the ICCs of both cost measures and “number of months” were poor.^45^

The reference sample’s test results of functional mobility and verbal fluency differed from previously reported reference values. The participants in the current study performed single-task TUG slightly slower across all age groups compared with previous research.^46–48^ By contrast, the median score for the verbal fluency test was 23.5 in our study, whereas a previous Swedish study found mean scores of 17.8 (SD = 5.7) for individuals without tertiary education and 20.6 (SD = 5.7) for individuals with tertiary education.^32^ The latter discrepancy may be due to a relatively high educational level in our sample.

The current reference values for TUGdt test variables suggested declining dt ability with age, which is in accordance with previous research.^7^ However, the lower limits for the number of animals and months were both unchanged across age groups among women. That is, regardless of age group, 97.5% of women named at least 6 animals and 4 months in correct reverse order, during TUGdt, and their corresponding time scores increased with age. When using reference values, it is important to consider that they indicate how to interpret TUGdt test results in relation to the performance of cognitively healthy individuals. Reference values should not be seen as cutoffs that define a “normal” performance, because an individual’s test result that is poorer than the reference value can be interpreted as a probability of 2.5% of random variability and absence of cognitive impairment. A deviating test result is therefore a reason for further investigation of the individual.

The excellent reliability indicated by the ICC estimates of TUGdt NA and MB time scores is in line with previous research on time scores or gait speed during different types of dt tests.^18–22^ Compared with time scores, the number of animals named as well as the number of months recited during TUGdt had lower reliability, a finding that is also consistent with previous research.^19^^,^^22^ A possible explanation as to why the time scores are more reliable than the verbal outcomes is that the mobility task is more automatized than the verbal tasks. Moreover, attention is a multifaceted cognitive construct, which may make cognitive performance unstable and more variable.^22^^,^^49^ This may have affected all variables that involved verbal performance. For the variables animals/10 s and months/10 s, the ICC estimates showed a fair to good level of reliability. These relatively low ICC estimates may be explained by a possible inflation in systematic error due to combining 2 variables.^19^ For a variable constructed as a ratio of 2 other variables, the reliability is lower for the ratio than for the nominator and the denominator separately, when these are positively correlated and their measurement errors are not correlated.^50^ For example, even though months/10 s is based on original single-task tests with high to excellent test–retest reliability (TUG^20^^,^^23^^,^^24^ and the months backward test^27^^,^^28^), it has relatively low reliability, which may result from both variations in attention and the merging of 2 variables. For the TUGdt cost measures, ICC estimates were poor, which is in accordance with previous research.^22^ Because these variables are also ratios, inflation in systematic error will once again be a factor in the estimates. However, the poor reliability for the cost measures was confirmed by SEM estimates, which suggested a wide within-participant variability, and by MDC, which showed that large differences between assessments could be expected by chance (24.06 for TUGdt NA cost and 30.66 for TUGdt MB cost).

The estimated ICCs included both random variability and putative systematic differences. The Bland–Altman plots of TUGdt MB time score and TUGdt NA animals/10 s both revealed systematic error. However, because the differences were numerically small and no other TUGdt variables showed significant differences between measurements, the errors may be seen as due to random error. Furthermore, 7 outliers were identified in the Bland–Altman plots of TUGdt NA and MB time scores, animals/10 s, and months/10 s. These participants behaved differently on 1 test occasion compared with the other. For the TUGdt NA test, the reasons for this were either laughter or the chair being accidently pushed before sitting down. Random errors of this kind during testing could be reduced by redoing the test when they occur. For the clinical use of TUGdt tests, an additional measurement could be considered when there are apparent disruptions of the performance. For TUGdt MB, distinct reasons for the outliers were not easy to identify because the verbal mistakes, hesitations, and slow gait speed producing outlying scores are all possible effects of dt interference.

In our ongoing UDDGait project, the target population consists of individuals with subjective and/or objective cognitive decline. It is not certain that the results of this study can be generalized to that population. Other research has shown excellent levels of reliability for time scores and step parameters during dt testing among individuals with mild cognitive impairment or dementia.^51–53^ However, the reliability of dt test outcomes may be affected negatively by cognitive impairment.^54^ As previously noted, the most robust TUGdt test outcome for predicting dementia incidence among individuals with mild or subjective cognitive impairment found in a previous UDDGait study was animals/10 s.^15^ In the current study, the MDC for animals/10 s showed that a change of at least 4 animals/10 s was required to distinguish a true change in performance. This points out that animals/10 s is not useful for, as an example, evaluating an exercise intervention. However, our main aim with the UDDGait TUGdt tests is prediction of conversion to dementia by a 1-time assessment. Given that animals/10 s has previously demonstrated good predictive capacity, despite the relatively low ICC estimates reported here, there is strong evidence that animals/10 s has the potential to be a useful dt test variable, even with the risk of regression dilution bias.^55^ This illustrates that interpretation of ICC estimates should not be considered as absolute evidence of a test’s usefulness but should include consideration of the clinical relevance of the results,^56^ in this case the variable’s predictive capacity.

This study has some limitations. The reference sample was recruited in a university city, and their relatively high educational level could have affected the results. However, a sensitivity analysis found negligible differences related to educational level. Also, generalizing our findings to populations who do not speak Swedish should be done with caution. The number of participants in our reliability sample was less than that recommended by COSMIN, but a sample of 43 is well above the minimal acceptable number of 30 participants.^57^ Another possible study limitation was that due to practical reasons, the number of days between test and retest was outside the set interval for some participants. However, our sensitivity analysis found no evidence that such deviations affected the results.

Our study also has several strengths, including the choice of statistical methods. The reference values were calculated by quantile regression, which is useful for understanding an outcome at its various quantiles and enables clinical comparisons.^58^ Additionally, for test–retest reliability, ICC, SEM, and MDC were used, supplemented by Bland–Altman plots, which are recommended methods for this purpose.^59^^,^^60^ Other strengths are related to the reliable TUGdt testing procedures, including standardized instructions and the recording of the verbal performance, which enabled a subsequent validation of the verbal results.^11^ Additionally, requirements for reliability studies,^29^ such as independent administrations, an appropriate time interval between test and retest, control of participants’ physical changes between test and retest, and similar test conditions, were all met.

In summary, we have established age- and sex-specific normative reference values for the UDDGait TUGdt test variables, potentially useful for clinical as well as research purposes. Our reliability analyses indicated excellent reliability for TUGdt time scores, with poor to good reliability for the other test variables. The reliability of the combined variables may have been affected negatively due to being constructed as ratios. Despite the reliability of animals/10 s being only fair to good, previous results regarding the variable’s predictive capacity suggest a potential usefulness for this test outcome. Ongoing and future UDDGait studies will examine the potential of TUGdt testing by establishing the test–retest reliability of TUGdt among individuals with cognitive impairment as well as continuing to investigate the predictive capacity of TUGdt testing over time.

## References

[1] World Health Organization . Dementia: a public health priority. 2012. Accessed 2 June, 2021. http://www.who.int/mental_health/publications/dementia_report_2012/en/.

[2] Laske C, Sohrabi HR, Frost SM, et al. Innovative diagnostic tools for early detection of Alzheimer's disease. Alzheimers Dement. 2015;11:561–578. 25443858 10.1016/j.jalz.2014.06.004

[3] Muir SW, Speechley M, Wells J, Borrie M, Gopaul K, Montero-Odasso M. Gait assessment in mild cognitive impairment and Alzheimer's disease: the effect of dual-task challenges across the cognitive spectrum. Gait Posture. 2012;35:96–100. 21940172 10.1016/j.gaitpost.2011.08.014

[4] MacAulay RK, Wagner MT, Szeles D, Milano NJ. Improving sensitivity to detect mild cognitive impairment: cognitive load dual-task gait speed assessment. J Int Neuropsychol Soc. 2017;23:493–501. 28413999 10.1017/S1355617717000261

[5] Cullen S, Borrie M, Carroll S, et al. Are cognitive subtypes associated with dual-task gait performance in a clinical setting? J Alzheimers Dis. 2019;71:S57–S64. 31322559 10.3233/JAD-181196

[6] Beurskens R, Bock O. Age-related deficits of dual-task walking: a review. Neural Plast. 2012;2012:131608. 22848845 10.1155/2012/131608 PMC3403123

[7] Brustio PR, Magistro D, Zecca M, Rabaglietti E, Liubicich ME. Age-related decrements in dual-task performance: comparison of different mobility and cognitive tasks. A cross sectional study. PLoS One. 2017;12:e0181698. 28732080 10.1371/journal.pone.0181698 PMC5521845

[8] Bahureksa L, Najafi B, Saleh A, et al. The impact of mild cognitive impairment on gait and balance: a systematic review and meta-analysis of studies using instrumented assessment. Gerontology. 2017;63:67–83. 27172932 10.1159/000445831 PMC5107359

[9] Perry RJ, Hodges JR. Attention and executive deficits in Alzheimer's disease. A critical review. Brain. 1999;122:383–404. 10094249 10.1093/brain/122.3.383

[10] Srygley JM, Mirelman A, Herman T, Giladi N, Hausdorff JM. When does walking alter thinking? Age and task associated findings. Brain Res. 2009;1253:92–99. 19084511 10.1016/j.brainres.2008.11.067 PMC2631095

[11] Cedervall Y, Stenberg AM, Åhman HB, et al. Timed up-and-go dual-task testing in the assessment of cognitive function: a mixed methods observational study for development of the UDDGait protocol. Int J Environ Res Public Health. 2020;17:1715. 10.3390/ijerph17051715 PMC7084863 32150995

[12] Podsiadlo D, Richardson S. The Timed “Up & Go”: a test of basic functional mobility for frail elderly persons. J Am Geriatr Soc. 1991;39:142–148. 1991946 10.1111/j.1532-5415.1991.tb01616.x

[13] Åhman HB, Giedraitis V, Cedervall Y, et al. Dual-task performance and neurodegeneration: correlations between timed up-and-go dual-task test outcomes and Alzheimer's disease cerebrospinal fluid biomarkers. J Alzheimers Dis. 2019;71:S75–s83. 31104024 10.3233/JAD-181265 PMC6839487

[14] Åhman HB, Cedervall Y, Kilander L, et al. Dual-task tests discriminate between dementia, mild cognitive impairment, subjective cognitive impairment, and healthy controls - a cross-sectional cohort study. BMC Geriatr. 2020;20:258. 32727472 10.1186/s12877-020-01645-1 PMC7392684

[15] Åhman HB, Berglund L, Cedervall Y, et al. Dual-task tests predict conversion to dementia-a prospective memory-clinic-based cohort study. Int J Environ Res Public Health. 2020;17:8129. 33153203 10.3390/ijerph17218129 PMC7662628

[16] Harris EK, Boyd JC. Statistical Bases of Reference Values in Laboratory Medicine. New York: Marcel Dekker, Inc; 1995.

[17] Bland JM, Altman DG. A note on the use of the intraclass correlation coefficient in the evaluation of agreement between two methods of measurement. Comput Biol Med. 1990;20:337–340. 2257734 10.1016/0010-4825(90)90013-f

[18] Hofheinz M, Schusterschitz C. Dual task interference in estimating the risk of falls and measuring change: a comparative, psychometric study of four measurements. Clin Rehabil. 2010;24:831–842. 20562166 10.1177/0269215510367993

[19] Muhaidat J, Kerr A, Evans JJ, Skelton DA. The test-retest reliability of gait-related dual task performance in community-dwelling fallers and non-fallers. Gait Posture. 2013;38:43–50. 23146196 10.1016/j.gaitpost.2012.10.011

[20] Smith E, Walsh L, Doyle J, Greene B, Blake C. The reliability of the quantitative timed up and go test (QTUG) measured over five consecutive days under single and dual-task conditions in community dwelling older adults. Gait Posture. 2016;43:239–244. 26526223 10.1016/j.gaitpost.2015.10.004

[21] McCulloch KL, Mercer V, Giuliani C, Marshall S. Development of a clinical measure of dual-task performance in walking: reliability and preliminary validity of the walking and remembering test. J Geriatr Phys Ther. 2009;32:2–9. 19856629 10.1519/00139143-200932010-00002

[22] Yang L, Liao LR, Lam FMH, He CQ, Pang MYC. Psychometric properties of dual-task balance assessments for older adults: a systematic review. Maturitas. 2015;80:359–369. 25618745 10.1016/j.maturitas.2015.01.001

[23] Donoghue OA, Savva GM, Börsch-Supan A, Kenny RA. Reliability, measurement error and minimum detectable change in mobility measures: a cohort study of community-dwelling adults aged 50 years and over in Ireland. BMJ Open. 2019;9:e030475. 10.1136/bmjopen-2019-030475 PMC6858113 31719075

[24] Nordin E, Rosendahl E, Lundin-Olsson L. Timed "up & go" test: reliability in older people dependent in activities of daily living--focus on cognitive state. Phys Ther. 2006;86:646–655. 16649889

[25] Bird CM, Papadopoulou K, Ricciardelli P, Rossor MN, Cipolotti L. Monitoring cognitive changes: psychometric properties of six cognitive tests. Br J Clin Psychol. 2004;43:197–210. 15169618 10.1348/014466504323088051

[26] Harrison JE, Buxton P, Husain M, Wise R. Short test of semantic and phonological fluency: normal performance, validity and test-retest reliability. Br J Clin Psychol. 2000;39:181–191. 10895361 10.1348/014466500163202

[27] Ostberg P, Hansson V, Haagg S. Adult norms and test-retest reliability for the months backward test: durational and response accuracy measures. Logoped Phoniatr Vocol. 2012;37:11–17. 21961729 10.3109/14015439.2011.614957

[28] Meagher J, Leonard M, Donoghue L, et al. Months backward test: a review of its use in clinical studies. World J Psychiatr. 2015;5:305–314. 26425444 10.5498/wjp.v5.i3.305 PMC4582306

[29] Mokkink LB . COSMIN Study Design Checklist for patient-reported outcome measurement instruments. 2020. Accessed November 27, 2020. https://www.cosmin.nl/wp-content/uploads/COSMIN-study-designing-checklist_final.pdf.

[30] COSMIN (COnsensus-based Standards for the selection of health Measurement INstruments) . COSMIN research projects. 2020. Accessed March 19, 2021. https://www.cosmin.nl/research-publications/.

[31] Folstein MF, Folstein SE, McHugh PR. "Mini-mental state". A practical method for grading the cognitive state of patients for the clinician. J Psychiatr Res. 1975;12:189–198. 1202204 10.1016/0022-3956(75)90026-6

[32] Tallberg IM, Ivachova E, Jones Tinghag K, Ostberg P. Swedish norms for word fluency tests: FAS, animals and verbs. Scand J Psychol. 2008;49:479–485. 18452499 10.1111/j.1467-9450.2008.00653.x

[33] Tombaugh TN . Trail making test a and B: normative data stratified by age and education. Arch Clin Neuropsychol. 2004;19:203–214. 15010086 10.1016/S0887-6177(03)00039-8

[34] Park J, Jeong E, Seomun G. The clock drawing test: a systematic review and meta-analysis of diagnostic accuracy. J Adv Nurs. 2018;74:2742–2754. 30047147 10.1111/jan.13810

[35] Almeida OP, Almeida SA. Short versions of the geriatric depression scale: a study of their validity for the diagnosis of a major depressive episode according to ICD-10 and DSM-IV. Int J Geriatr Psychiatry. 1999;14:858–865. 10521885 10.1002/(sici)1099-1166(199910)14:10<858::aid-gps35>3.0.co;2-8

[36] Aberg AC, Lindmark B, Lithell H. Development and reliability of the general motor function assessment scale (GMF)--a performance-based measure of function-related dependence, pain and insecurity. Disabil Rehabil. 2003;25:462–472. 12745941 10.1080/0963828031000069762

[37] Bohannon RW, Larkin PA, Cook AC, Gear J, Singer J. Decrease in timed balance test scores with aging. Phy Ther. 1984;64:1067–1070. 10.1093/ptj/64.7.1067 6739548

[38] Bohannon RW . Test-retest reliability of measurements of hand-grip strength obtained by dynamometry from older adults: a systematic review of research in the PubMed database. J Frailty Aging. 2017;6:83–87. 28555708 10.14283/jfa.2017.8

[39] Wei Y, Pere A, Koenker R, He X. Quantile regression methods for reference growth charts. Stat Med. 2006;25:1369–1382. 16143984 10.1002/sim.2271

[40] Lê Cook B, Manning WG. Thinking beyond the mean: a practical guide for using quantile regression methods for health services research. Shanghai Arch Psychiatry. 2013;25:55–59. 24948867 10.3969/j.issn.1002-0829.2013.01.011 PMC4054530

[41] Koo TK, Li MY. A guideline of selecting and reporting intraclass correlation coefficients for reliability research. J Chiropr Med. 2016;15:155–163. 27330520 10.1016/j.jcm.2016.02.012 PMC4913118

[42] Chernick R . Bootstrap Methods: A Practitioner's Guide. New York, NY: John Wiley & Sons, Inc; 1999.

[43] Bland JM, Altman DG. Measuring agreement in method comparison studies. Stat Methods Med Res. 1999;8:135–160. 10501650 10.1177/096228029900800204

[44] R Core Team. R: A language and environment for statistical computing. R Foundation for Statistical Computing, Vienna, Austria. 2020. Accessed 2 June 2021. https://www.R-project.org/

[45] Rosner BA . Fundamentals of Biostatistics. Boston, MA: Thomson-Brooks/Cole; 2006.

[46] Bohannon RW . Reference values for the timed up and go test: a descriptive meta-analysis. J Geriatr Phys Ther. 2006;29:64–68. 16914068 10.1519/00139143-200608000-00004

[47] Steffen TM, Hacker TA, Mollinger L. Age- and gender-related test performance in community-dwelling elderly people: six-minute walk test, berg balance scale, timed up & go test, and gait speeds. Phys Ther. 2002;82:128–137. 11856064 10.1093/ptj/82.2.128

[48] Pondal M, del Ser T. Normative data and determinants for the timed "up and go" test in a population-based sample of elderly individuals without gait disturbances. J Geriatr Phys Ther. 2008;31:57–63. 19856551 10.1519/00139143-200831020-00004

[49] Yang L, He C, Pang MY. Reliability and validity of dual-task mobility assessments in people with chronic stroke. PLoS One. 2016;11:e0147833. 26808662 10.1371/journal.pone.0147833 PMC4726712

[50] Nordhamn K, Södergren E, Olsson E, Karlström B, Vessby B, Berglund L. Reliability of anthropometric measurements in overweight and lean subjects: consequences for correlations between anthropometric and other variables. IJO. 2000;24:652–657. 10.1038/sj.ijo.0801216 10849590

[51] Pettersson AF, Olsson E, Wahlund LO. Effect of divided attention on gait in subjects with and without cognitive impairment. J Geriatr Psychiatry Neurol. 2007;20:58–62. 17341772 10.1177/0891988706293528

[52] Montero-Odasso M, Casas A, Hansen KT, et al. Quantitative gait analysis under dual-task in older people with mild cognitive impairment: a reliability study. J Neuroeng Rehabil. 2009;6:35. 19772593 10.1186/1743-0003-6-35 PMC2754991

[53] Lemke NC, Wiloth S, Werner C, Hauer K. Validity, test-retest reliability, sensitivity to change and feasibility of motor-cognitive dual task assessments in patients with dementia. Arch Gerontol Geriatr. 2017;70:169–179. 28182895 10.1016/j.archger.2017.01.016

[54] Venema DM, Hansen H, High R, Goetsch T, Siu KC. Minimal detectable change in dual-task cost for older adults with and without cognitive impairment. J Geriatr Phys Ther. 2019;42:E32–E38. 10.1519/JPT.0000000000000194 29864048

[55] Berglund L . Regression dilution bias: tools for correction methods and sample size calculation. Ups J Med Sci. 2012;117:279–283. 22401135 10.3109/03009734.2012.668143 PMC3410287

[56] Kottner J, Audigé L, Brorson S, et al. Guidelines for reporting reliability and agreement studies (GRRAS) were proposed. J Clin Epidemiol. 2011;64:96–106. 21130355 10.1016/j.jclinepi.2010.03.002

[57] Terwee CB, Bot SD, de Boer MR, et al. Quality criteria were proposed for measurement properties of health status questionnaires. J Clin Epidemiol. 2007;60:34–42. 17161752 10.1016/j.jclinepi.2006.03.012

[58] Staffa SJ, Kohane DS, Zurakowski D. Quantile regression and its applications: a primer for anesthesiologists. Anesth Analg. 2019;128:820–830. 30649075 10.1213/ANE.0000000000004017

[59] Giavarina D . Understanding Bland Altman analysis. Biochem Med (Zagreb). 2015;25:141–151. 26110027 10.11613/BM.2015.015 PMC4470095

[60] Mokkink LB, Terwee CB, Patrick DL, et al. The COSMIN study reached international consensus on taxonomy, terminology, and definitions of measurement properties for health-related patient-reported outcomes. J Clin Epidemiol. 2010;63:737–745. 20494804 10.1016/j.jclinepi.2010.02.006

